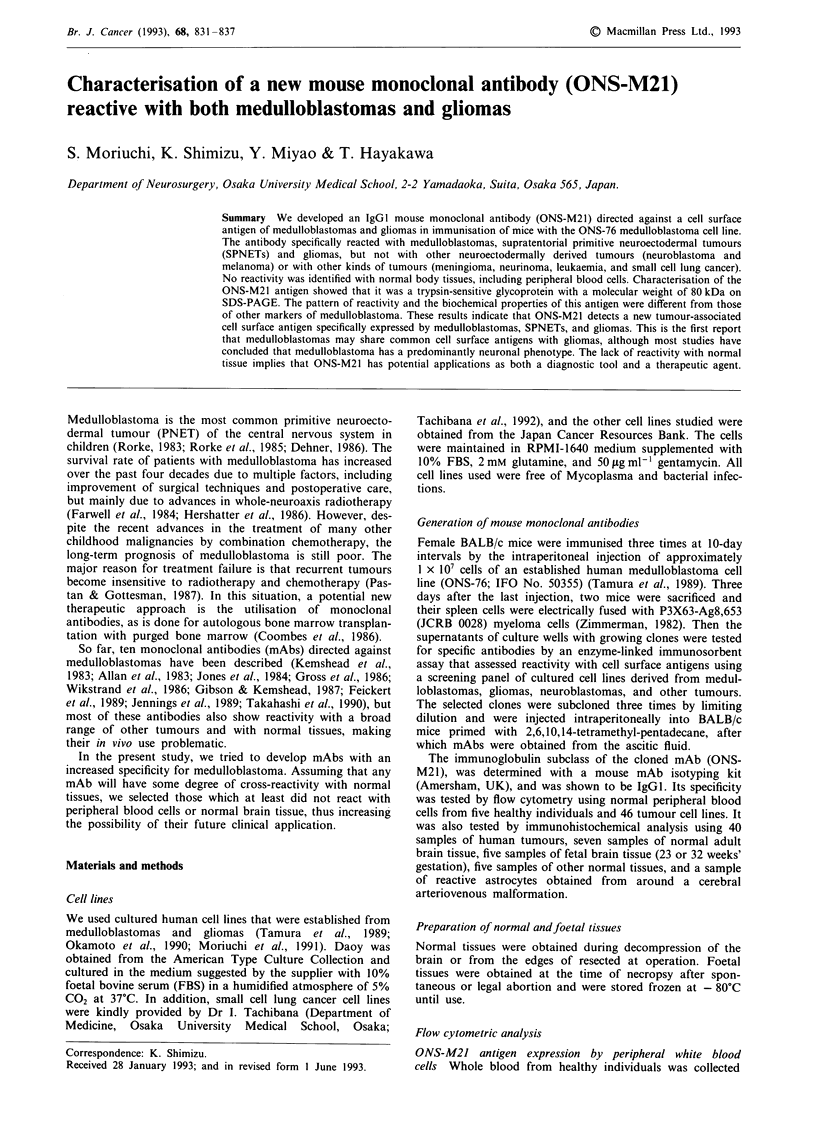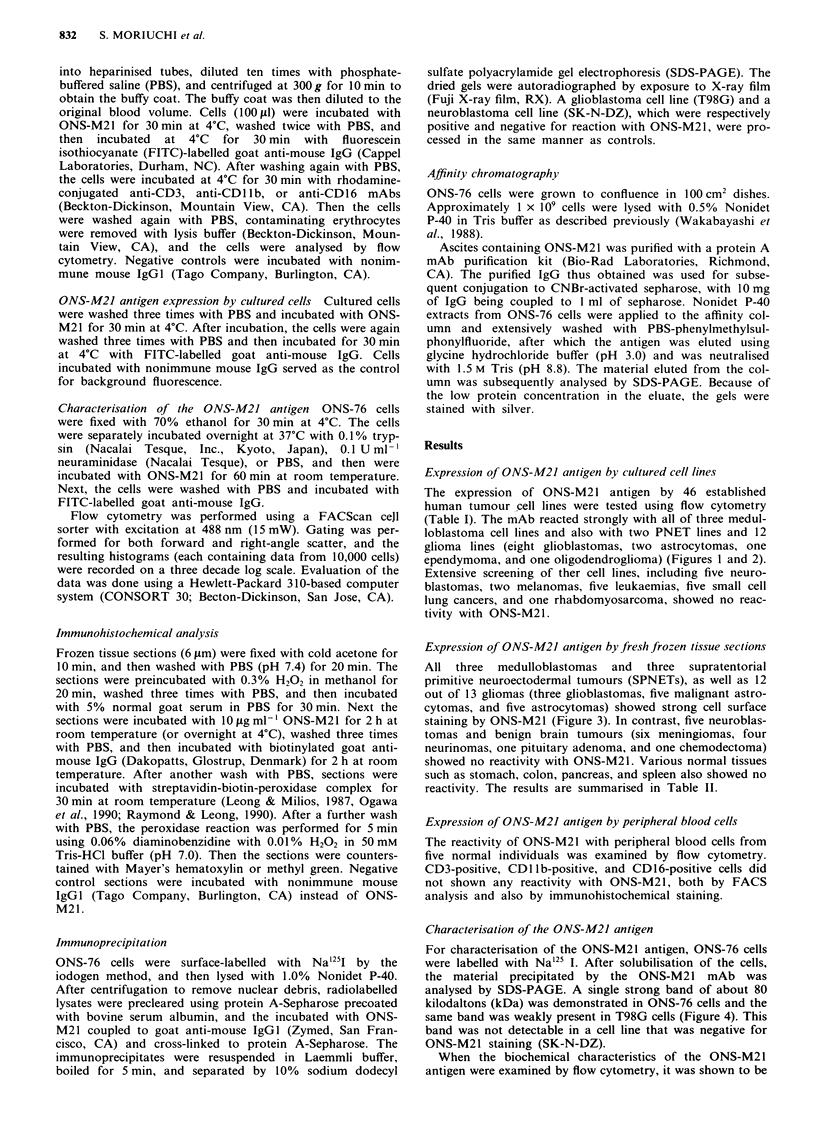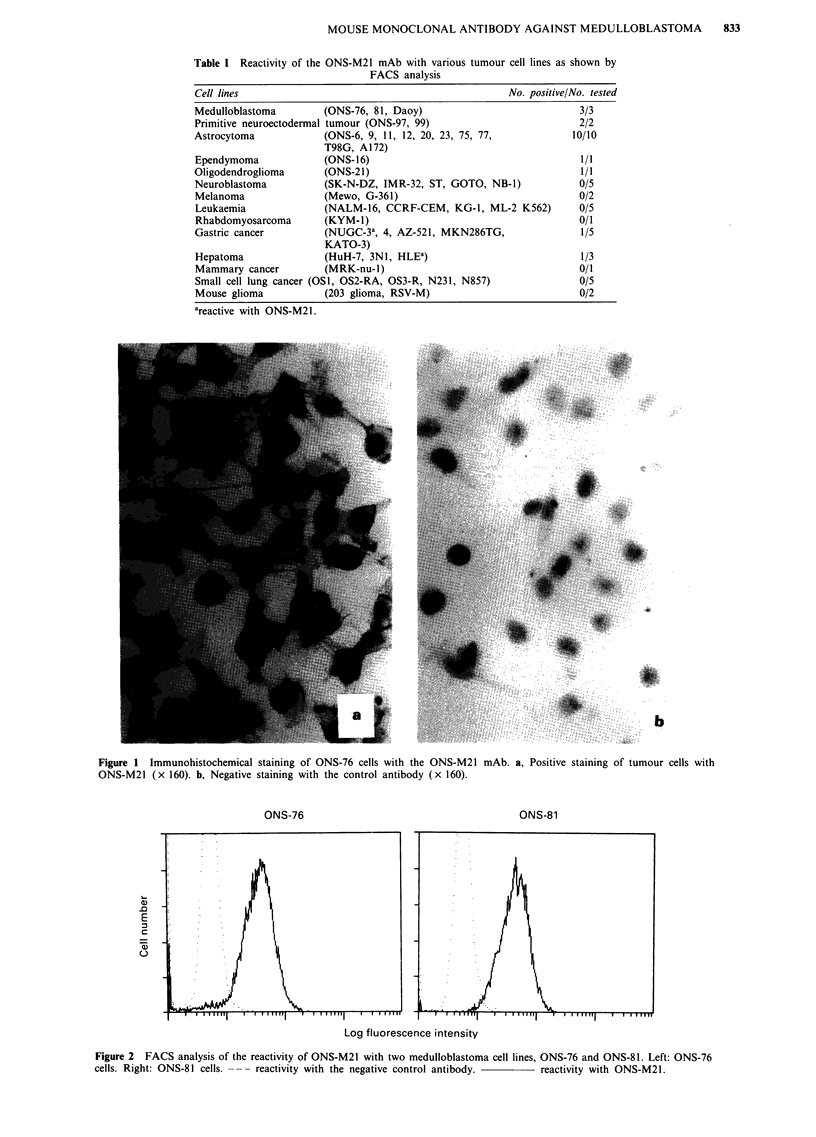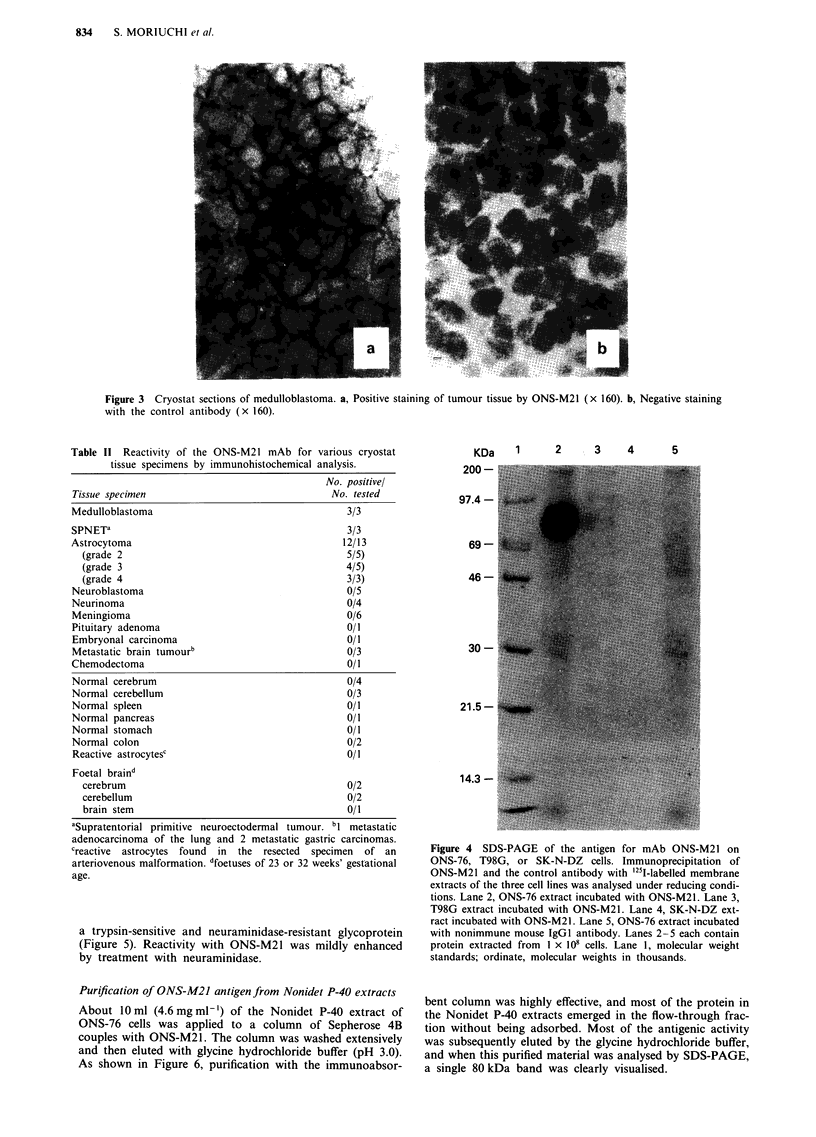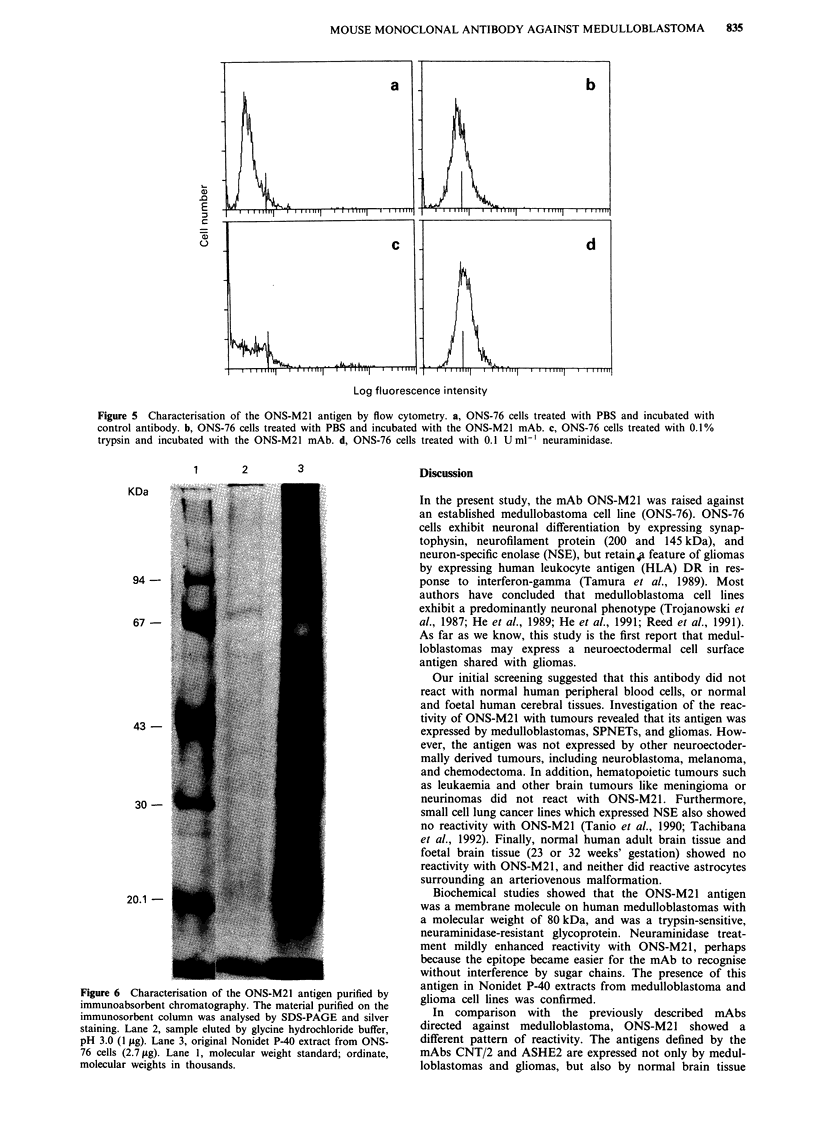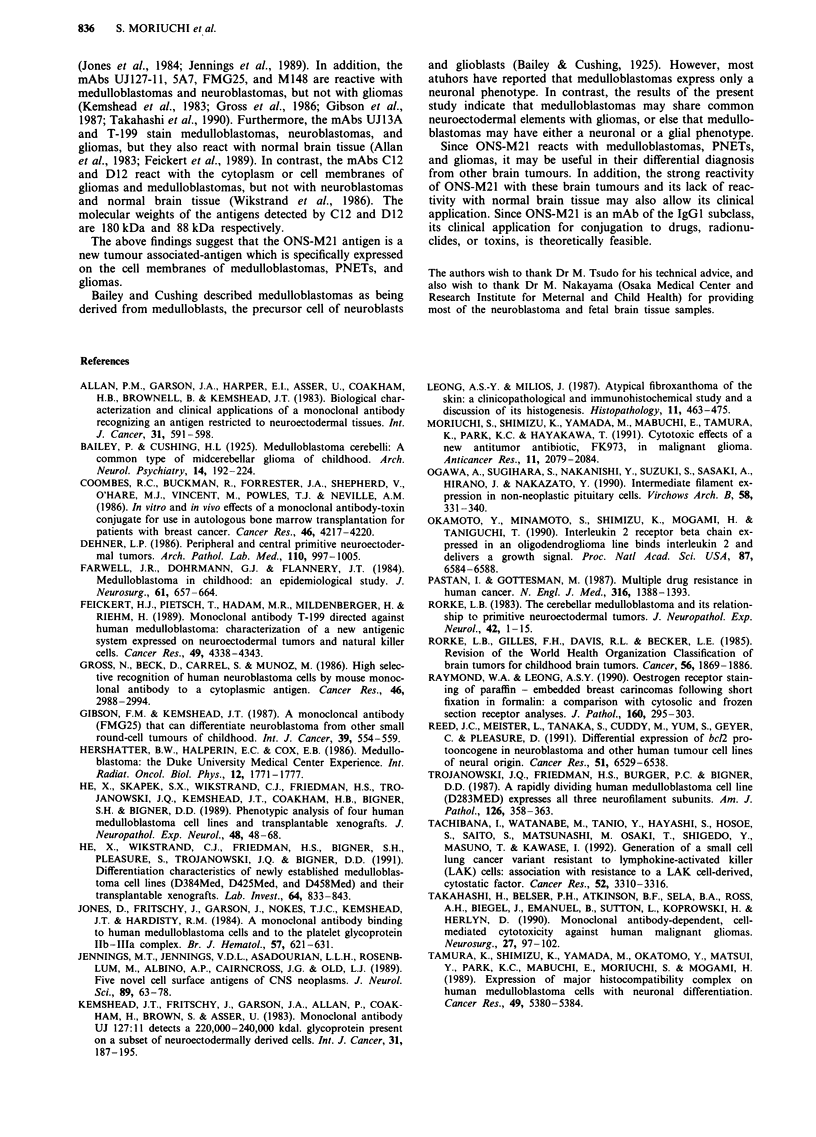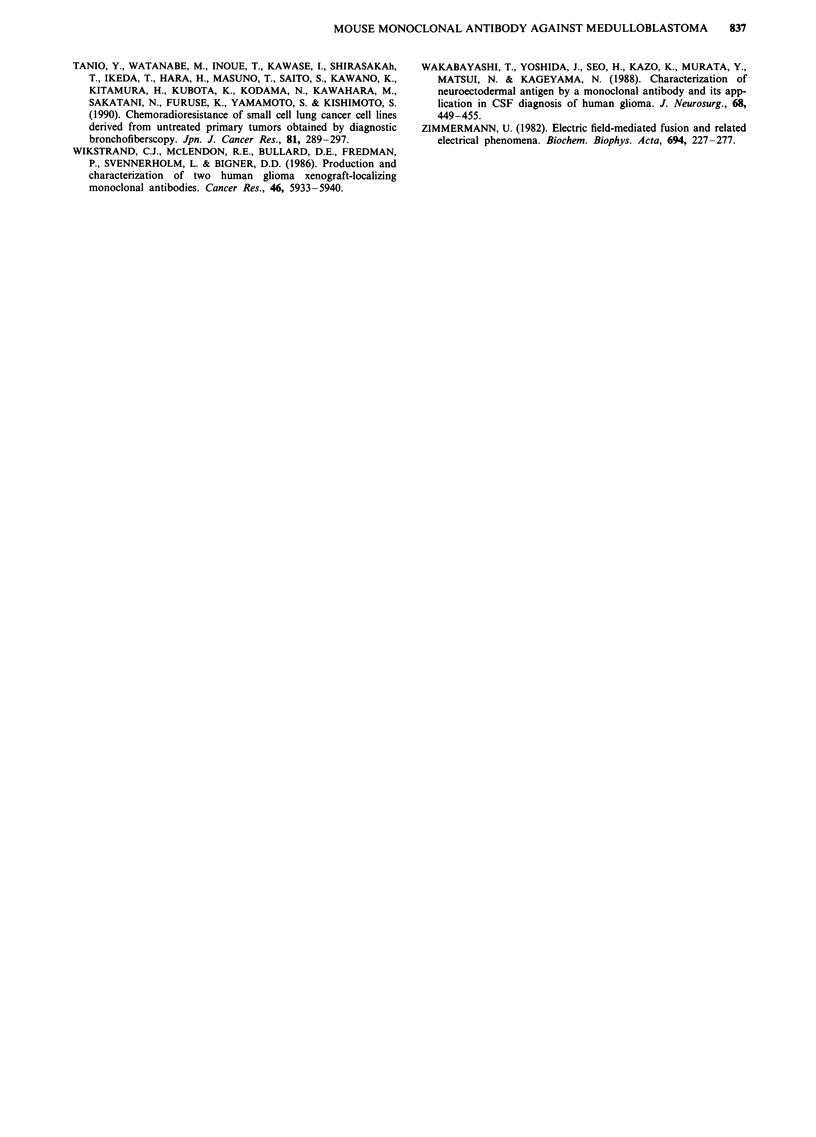# Characterisation of a new mouse monoclonal antibody (ONS-M21) reactive with both medulloblastomas and gliomas.

**DOI:** 10.1038/bjc.1993.441

**Published:** 1993-11

**Authors:** S. Moriuchi, K. Shimizu, Y. Miyao, T. Hayakawa

**Affiliations:** Department of Neurosurgery, Osaka University Medical School, Japan.

## Abstract

**Images:**


 © Macmillan Press Ltd., 1993

### Characterisation of a new mouse monoclonal antibody (ONS-M21) reactive with both medulloblastomas and gliomas

S. Moriuchi, K. Shimizu, Y. Miyao & T. Hayakawa

*Department of Neurosurgery, Osaka University Medical School, 2-2 Yamadaoka, Suita, Osaka 565, Japan.*

**Summary** We developed an IgG1 mouse monoclonal antibody (ONS-M21) directed against a cell surface antigen of medulloblastomas and gliomas in immunisation of mice with the ONS-76 medulloblastoma cell line. The antibody specifically reacted with medulloblastomas, supratentorial primitive neuroectodermal tumours (SPNETs) and gliomas, but not with other neuroectodermally derived tumours (neuroblastoma and melanoma) or with other kinds of tumours (meningioma, neurinoma, leukaemia, and small cell lung cancer). No reactivity was identified with normal body tissues, including peripheral blood cells. Characterisation of the ONS-M21 antigen showed that it was a trypsin-sensitive glycoprotein with a molecular weight of 80 kDa on SDS-PAGE. The pattern of reactivity and the biochemical properties of this antigen were different from those of other markers of medulloblastoma. These results indicate that ONS-M21 detects a new tumour-associated cell surface antigen specifically expressed by medulloblastomas, SPNETs, and gliomas. This is the first report that medulloblastomas may share common cell surface antigens with gliomas, although most studies have concluded that medulloblastoma has a predominantly neuronal phenotype. The lack of reactivity with normal tissue implies that ONS-M21 has potential applications as both a diagnostic tool and a therapeutic agent.

Medulloblastoma is the most common primitive neuroectodermal tumour (PNET) of the central nervous system in children (Rorke, 1983; Rorke *et al.*, 1985; Dehner, 1986). The survival rate of patients with medulloblastoma has increased over the past four decades due to multiple factors, including improvement of surgical techniques and postoperative care, but mainly due to advances in whole-neuroaxis radiotherapy (Farwell *et al.*, 1984; Hershatter *et al.*, 1986). However, despite the recent advances in the treatment of many other childhood malignancies by combination chemotherapy, the long-term prognosis of medulloblastoma is still poor. The major reason for treatment failure is that recurrent tumours become insensitive to radiotherapy and chemotherapy (Pastan & Gottesman, 1987). In this situation, a potential new therapeutic approach is the utilisation of monoclonal antibodies, as is done for autologous bone marrow transplantation with purged bone marrow (Coombes *et al.*, 1986).

So far, ten monoclonal antibodies (mAbs) directed against medulloblastomas have been described (Kemshead *et al.*, 1983; Allan *et al.*, 1983; Jones *et al.*, 1984; Gross *et al.*, 1986; Wikstrand *et al.*, 1986; Gibson & Kemshead, 1987; Feickert *et al.*, 1989; Jennings *et al.*, 1989; Takahashi *et al.*, 1990), but most of these antibodies also show reactivity with a broad range of other tumours and with normal tissues, making their *in vivo* use problematic.

In the present study, we tried to develop mAbs with an increased specificity for medulloblastoma. Assuming that any mAb will have some degree of cross-reactivity with normal tissues, we selected those which at least did not react with peripheral blood cells or normal brain tissue, thus increasing the possibility of their future clinical application.

### Materials and methods

### *Cell lines*

We used cultured human cell lines that were established from medulloblastomas and gliomas (Tamura *et al.*, 1989; Okamoto *et al.*, 1990; Moriuchi *et al.*, 1991). Daoy was obtained from the American Type Culture Collection and cultured in the medium suggested by the supplier with 10% foetal bovine serum (FBS) in a humidified atmosphere of 5% $CO_2$ at 37°C. In addition, small cell lung cancer cell lines were kindly provided by Dr I. Tachibana (Department of Medicine, Osaka University Medical School, Osaka; Tachibana *et al.*, 1992), and the other cell lines studied were obtained from the Japan Cancer Resources Bank. The cells were maintained in RPMI-1640 medium supplemented with 10% FBS, 2 mM glutamine, and 50 $\mu g\ ml^{-1}$ gentamycin. All cell lines used were free of Mycoplasma and bacterial infections.

### *Generation of mouse monoclonal antibodies*

Female BALB/c mice were immunised three times at 10-day intervals by the intraperitoneal injection of approximately $1 \times 10^7$ cells of an established human medulloblastoma cell line (ONS-76; IFO No. 50355) (Tamura *et al.*, 1989). Three days after the last injection, two mice were sacrificed and their spleen cells were electrically fused with P3X63-Ag8,653 (JCRB 0028) myeloma cells (Zimmerman, 1982). Then the supernatants of culture wells with growing clones were tested for specific antibodies by an enzyme-linked immunosorbent assay that assessed reactivity with cell surface antigens using a screening panel of cultured cell lines derived from medulloblastomas, gliomas, neuroblastomas, and other tumours. The selected clones were subcloned three times by limiting dilution and were injected intraperitoneally into BALB/c mice primed with 2,6,10,14-tetramethyl-pentadecane, after which mAbs were obtained from the ascitic fluid.

The immunoglobulin subclass of the cloned mAb (ONS-M21), was determined with a mouse mAb isotyping kit (Amersham, UK), and was shown to be IgG1. Its specificity was tested by flow cytometry using normal peripheral blood cells from five healthy individuals and 46 tumour cell lines. It was also tested by immunohistochemical analysis using 40 samples of human tumours, seven samples of normal adult brain tissue, five samples of fetal brain tissue (23 or 32 weeks' gestation), five samples of other normal tissues, and a sample of reactive astrocytes obtained from around a cerebral arteriovenous malformation.

### *Preparation of normal and foetal tissues*

Normal tissues were obtained during decompression of the brain or from the edges of resected at operation. Foetal tissues were obtained at the time of necropsy after spontaneous or legal abortion and were stored frozen at − 80°C until use.

### *Flow cytometric analysis*

*ONS-M21 antigen expression by peripheral white blood cells* Whole blood from healthy individuals was collected

Correspondence: K. Shimizu.
Received 28 January 1993; and in revised form 1 June 1993.

into heparinised tubes, diluted ten times with phosphate-buffered saline (PBS), and centrifuged at 300 *g* for 10 min to obtain the buffy coat. The buffy coat was then diluted to the original blood volume. Cells (100 μl) were incubated with ONS-M21 for 30 min at 4°C, washed twice with PBS, and then incubated at 4°C for 30 min with fluorescein isothiocyanate (FITC)-labelled goat anti-mouse IgG (Cappel Laboratories, Durham, NC). After washing again with PBS, the cells were incubated at 4°C for 30 min with rhodamine-conjugated anti-CD3, anti-CD11b, or anti-CD16 mAbs (Beckton-Dickinson, Mountain View, CA). Then the cells were washed again with PBS, contaminating erythrocytes were removed with lysis buffer (Beckton-Dickinson, Mountain View, CA), and the cells were analysed by flow cytometry. Negative controls were incubated with nonimmune mouse IgG1 (Tago Company, Burlington, CA).

*ONS-M21 antigen expression by cultured cells* Cultured cells were washed three times with PBS and incubated with ONS-M21 for 30 min at 4°C. After incubation, the cells were again washed three times with PBS and then incubated for 30 min at 4°C with FITC-labelled goat anti-mouse IgG. Cells incubated with nonimmune mouse IgG served as the control for background fluorescence.

*Characterisation of the ONS-M21 antigen* ONS-76 cells were fixed with 70% ethanol for 30 min at 4°C. The cells were separately incubated overnight at 37°C with 0.1% trypsin (Nacalai Tesque, Inc., Kyoto, Japan), 0.1 U $ml^{-1}$ neuraminidase (Nacalai Tesque), or PBS, and then were incubated with ONS-M21 for 60 min at room temperature. Next, the cells were washed with PBS and incubated with FITC-labelled goat anti-mouse IgG.

Flow cytometry was performed using a FACScan cell sorter with excitation at 488 nm (15 mW). Gating was performed for both forward and right-angle scatter, and the resulting histograms (each containing data from 10,000 cells) were recorded on a three decade log scale. Evaluation of the data was done using a Hewlett-Packard 310-based computer system (CONSORT 30; Becton-Dickinson, San Jose, CA).

### *Immunohistochemical analysis*

Frozen tissue sections (6 μm) were fixed with cold acetone for 10 min, and then washed with PBS (pH 7.4) for 20 min. The sections were preincubated with 0.3% $H_2O_2$ in methanol for 20 min, washed three times with PBS, and then incubated with 5% normal goat serum in PBS for 30 min. Next the sections were incubated with 10 μg $ml^{-1}$ ONS-M21 for 2 h at room temperature (or overnight at 4°C), washed three times with PBS, and then incubated with biotinylated goat anti-mouse IgG (Dakopatts, Glostrup, Denmark) for 2 h at room temperature. After another wash with PBS, sections were incubated with streptavidin-biotin-peroxidase complex for 30 min at room temperature (Leong & Milios, 1987, Ogawa *et al.*, 1990; Raymond & Leong, 1990). After a further wash with PBS, the peroxidase reaction was performed for 5 min using 0.06% diaminobenzidine with 0.01% $H_2O_2$ in 50 mM Tris-HCl buffer (pH 7.0). Then the sections were counterstained with Mayer's hematoxylin or methyl green. Negative control sections were incubated with nonimmune mouse IgG1 (Tago Company, Burlington, CA) instead of ONS-M21.

### *Immunoprecipitation*

ONS-76 cells were surface-labelled with $Na^{125}I$ by the iodogen method, and then lysed with 1.0% Nonidet P-40. After centrifugation to remove nuclear debris, radiolabelled lysates were precleared using protein A-Sepharose precoated with bovine serum albumin, and the incubated with ONS-M21 coupled to goat anti-mouse IgG1 (Zymed, San Francisco, CA) and cross-linked to protein A-Sepharose. The immunoprecipitates were resuspended in Laemmli buffer, boiled for 5 min, and separated by 10% sodium dodecyl sulfate polyacrylamide gel electrophoresis (SDS-PAGE). The dried gels were autoradiographed by exposure to X-ray film (Fuji X-ray film, RX). A glioblastoma cell line (T98G) and a neuroblastoma cell line (SK-N-DZ), which were respectively positive and negative for reaction with ONS-M21, were processed in the same manner as controls.

### *Affinity chromatography*

ONS-76 cells were grown to confluence in 100 $cm^2$ dishes. Approximately $1 \times 10^9$ cells were lysed with 0.5% Nonidet P-40 in Tris buffer as described previously (Wakabayashi *et al.*, 1988).

Ascites containing ONS-M21 was purified with a protein A mAb purification kit (Bio-Rad Laboratories, Richmond, CA). The purified IgG thus obtained was used for subsequent conjugation to CNBr-activated sepharose, with 10 mg of IgG being coupled to 1 ml of sepharose. Nonidet P-40 extracts from ONS-76 cells were applied to the affinity column and extensively washed with PBS-phenylmethylsulphonylfluoride, after which the antigen was eluted using glycine hydrochloride buffer (pH 3.0) and was neutralised with 1.5 M Tris (pH 8.8). The material eluted from the column was subsequently analysed by SDS-PAGE. Because of the low protein concentration in the eluate, the gels were stained with silver.

### Results

### *Expression of ONS-M21 antigen by cultured cell lines*

The expression of ONS-M21 antigen by 46 established human tumour cell lines were tested using flow cytometry (Table I). The mAb reacted strongly with all of three medulloblastoma cell lines and also with two PNET lines and 12 glioma lines (eight glioblastomas, two astrocytomas, one ependymoma, and one oligodendroglioma) (Figures 1 and 2). Extensive screening of ther cell lines, including five neuroblastomas, two melanomas, five leukaemias, five small cell lung cancers, and one rhabdomyosarcoma, showed no reactivity with ONS-M21.

### *Expression of ONS-M21 antigen by fresh frozen tissue sections*

All three medulloblastomas and three supratentorial primitive neuroectodermal tumours (SPNETs), as well as 12 out of 13 gliomas (three glioblastomas, five malignant astrocytomas, and five astrocytomas) showed strong cell surface staining by ONS-M21 (Figure 3). In contrast, five neuroblastomas and benign brain tumours (six meningiomas, four neurinomas, one pituitary adenoma, and one chemodectoma) showed no reactivity with ONS-M21. Various normal tissues such as stomach, colon, pancreas, and spleen also showed no reactivity. The results are summarised in Table II.

### *Expression of ONS-M21 antigen by peripheral blood cells*

The reactivity of ONS-M21 with peripheral blood cells from five normal individuals was examined by flow cytometry. CD3-positive, CD11b-positive, and CD16-positive cells did not shown any reactivity with ONS-M21, both by FACS analysis and also by immunohistochemical staining.

### *Characterisation of the ONS-M21 antigen*

For characterisation of the ONS-M21 antigen, ONS-76 cells were labelled with $Na^{125}$ I. After solubilisation of the cells, the material precipitated by the ONS-M21 mAb was analysed by SDS-PAGE. A single strong band of about 80 kilodaltons (kDa) was demonstrated in ONS-76 cells and the same band was weakly present in T98G cells (Figure 4). This band was not detectable in a cell line that was negative for ONS-M21 staining (SK-N-DZ).

When the biochemical characteristics of the ONS-M21 antigen were examined by flow cytometry, it was shown to be

**Table I** Reactivity of the ONS-M21 mAb with various tumour cell lines as shown by FACS analysis

| *Cell lines* | | *No. positive/No. tested* |
|---|---|---|
| Medulloblastoma | (ONS-76, 81, Daoy) | 3/3 |
| Primitive neuroectodermal tumour | (ONS-97, 99) | 2/2 |
| Astrocytoma | (ONS-6, 9, 11, 12, 20, 23, 75, 77, T98G, A172) | 10/10 |
| Ependymoma | (ONS-16) | 1/1 |
| Oligodendroglioma | (ONS-21) | 1/1 |
| Neuroblastoma | (SK-N-DZ, IMR-32, ST, GOTO, NB-1) | 0/5 |
| Melanoma | (Mewo, G-361) | 0/2 |
| Leukaemia | (NALM-16, CCRF-CEM, KG-1, ML-2 K562) | 0/5 |
| Rhabdomyosarcoma | (KYM-1) | 0/1 |
| Gastric cancer | (NUGC-3[a], 4, AZ-521, MKN286TG, KATO-3) | 1/5 |
| Hepatoma | (HuH-7, 3N1, HLE[a]) | 1/3 |
| Mammary cancer | (MRK-nu-1) | 0/1 |
| Small cell lung cancer | (OS1, OS2-RA, OS3-R, N231, N857) | 0/5 |
| Mouse glioma | (203 glioma, RSV-M) | 0/2 |

[a]reactive with ONS-M21.

**Figure 1** Immunohistochemical staining of ONS-76 cells with the ONS-M21 mAb. **a**, Positive staining of tumour cells with ONS-M21 (× 160). **b**, Negative staining with the control antibody (× 160).

**Figure 2** FACS analysis of the reactivity of ONS-M21 with two medulloblastoma cell lines, ONS-76 and ONS-81. Left: ONS-76 cells. Right: ONS-81 cells. --- reactivity with the negative control antibody. ——— reactivity with ONS-M21.

**Figure 3** Cryostat sections of medulloblastoma. **a**, Positive staining of tumour tissue by ONS-M21 (× 160). **b**, Negative staining with the control antibody (× 160).

**Table II** Reactivity of the ONS-M21 mAb for various cryostat tissue specimens by immunohistochemical analysis.

| *Tissue specimen* | *No. positive/ No. tested* |
|---|---|
| Medulloblastoma | 3/3 |
| SPNET[a] | 3/3 |
| Astrocytoma | 12/13 |
| (grade 2 | 5/5) |
| (grade 3 | 4/5) |
| (grade 4 | 3/3) |
| Neuroblastoma | 0/5 |
| Neurinoma | 0/4 |
| Meningioma | 0/6 |
| Pituitary adenoma | 0/1 |
| Embryonal carcinoma | 0/1 |
| Metastatic brain tumour[b] | 0/3 |
| Chemodectoma | 0/1 |
| Normal cerebrum | 0/4 |
| Normal cerebellum | 0/3 |
| Normal spleen | 0/1 |
| Normal pancreas | 0/1 |
| Normal stomach | 0/1 |
| Normal colon | 0/2 |
| Reactive astrocytes[c] | 0/1 |
| Foetal brain[d] | |
| cerebrum | 0/2 |
| cerebellum | 0/2 |
| brain stem | 0/1 |

[a]Supratentorial primitive neuroectodermal tumour. [b]1 metastatic adenocarcinoma of the lung and 2 metastatic gastric carcinomas. [c]reactive astrocytes found in the resected specimen of an arteriovenous malformation. [d]foetuses of 23 or 32 weeks' gestational age.

**Figure 4** SDS-PAGE of the antigen for mAb ONS-M21 on ONS-76, T98G, or SK-N-DZ cells. Immunoprecipitation of ONS-M21 and the control antibody with $^{125}$I-labelled membrane extracts of the three cell lines was analysed under reducing conditions. Lane 2, ONS-76 extract incubated with ONS-M21. Lane 3, T98G extract incubated with ONS-M21. Lane 4, SK-N-DZ extract incubated with ONS-M21. Lane 5, ONS-76 extract incubated with nonimmune mouse IgG1 antibody. Lanes 2–5 each contain protein extracted from 1 × $10^8$ cells. Lane 1, molecular weight standards; ordinate, molecular weights in thousands.

a trypsin-sensitive and neuraminidase-resistant glycoprotein (Figure 5). Reactivity with ONS-M21 was mildly enhanced by treatment with neuraminidase.

*Purification of ONS-M21 antigen from Nonidet P-40 extracts*

About 10 ml (4.6 mg $ml^{-1}$) of the Nonidet P-40 extract of ONS-76 cells was applied to a column of Sepherose 4B couples with ONS-M21. The column was washed extensively and then eluted with glycine hydrochloride buffer (pH 3.0). As shown in Figure 6, purification with the immunoabsorbent column was highly effective, and most of the protein in the Nonidet P-40 extracts emerged in the flow-through fraction without being adsorbed. Most of the antigenic activity was subsequently eluted by the glycine hydrochloride buffer, and when this purified material was analysed by SDS-PAGE, a single 80 kDa band was clearly visualised.

**Figure 5** Characterisation of the ONS-M21 antigen by flow cytometry. **a**, ONS-76 cells treated with PBS and incubated with control antibody. **b**, ONS-76 cells treated with PBS and incubated with the ONS-M21 mAb. **c**, ONS-76 cells treated with 0.1% trypsin and incubated with the ONS-M21 mAb. **d**, ONS-76 cells treated with 0.1 U $ml^{-1}$ neuraminidase.

**Figure 6** Characterisation of the ONS-M21 antigen purified by immunoabsorbent chromatography. The material purified on the immunosorbent column was analysed by SDS-PAGE and silver staining. Lane 2, sample eluted by glycine hydrochloride buffer, pH 3.0 (1 µg). Lane 3, original Nonidet P-40 extract from ONS-76 cells (2.7 µg). Lane 1, molecular weight standard; ordinate, molecular weights in thousands.

### Discussion

In the present study, the mAb ONS-M21 was raised against an established medullobastoma cell line (ONS-76). ONS-76 cells exhibit neuronal differentiation by expressing synaptophysin, neurofilament protein (200 and 145 kDa), and neuron-specific enolase (NSE), but retain a feature of gliomas by expressing human leukocyte antigen (HLA) DR in response to interferon-gamma (Tamura *et al.*, 1989). Most authors have concluded that medulloblastoma cell lines exhibit a predominantly neuronal phenotype (Trojanowski *et al.*, 1987; He *et al.*, 1989; He *et al.*, 1991; Reed *et al.*, 1991). As far as we know, this study is the first report that medulloblastomas may express a neuroectodermal cell surface antigen shared with gliomas.

Our initial screening suggested that this antibody did not react with normal human peripheral blood cells, or normal and foetal human cerebral tissues. Investigation of the reactivity of ONS-M21 with tumours revealed that its antigen was expressed by medulloblastomas, SPNETs, and gliomas. However, the antigen was not expressed by other neuroectodermally derived tumours, including neuroblastoma, melanoma, and chemodectoma. In addition, hematopoietic tumours such as leukaemia and other brain tumours like meningioma or neurinomas did not react with ONS-M21. Furthermore, small cell lung cancer lines which expressed NSE also showed no reactivity with ONS-M21 (Tanio *et al.*, 1990; Tachibana *et al.*, 1992). Finally, normal human adult brain tissue and foetal brain tissue (23 or 32 weeks' gestation) showed no reactivity with ONS-M21, and neither did reactive astrocytes surrounding an arteriovenous malformation.

Biochemical studies showed that the ONS-M21 antigen was a membrane molecule on human medulloblastomas with a molecular weight of 80 kDa, and was a trypsin-sensitive, neuraminidase-resistant glycoprotein. Neuraminidase treatment mildly enhanced reactivity with ONS-M21, perhaps because the epitope became easier for the mAb to recognise without interference by sugar chains. The presence of this antigen in Nonidet P-40 extracts from medulloblastoma and glioma cell lines was confirmed.

In comparison with the previously described mAbs directed against medulloblastoma, ONS-M21 showed a different pattern of reactivity. The antigens defined by the mAbs CNT/2 and ASHE2 are expressed not only by medulloblastomas and gliomas, but also by normal brain tissue

(Jones et al., 1984; Jennings et al., 1989). In addition, the mAbs UJ127-11, 5A7, FMG25, and M148 are reactive with medulloblastomas and neuroblastomas, but not with gliomas (Kemshead et al., 1983; Gross et al., 1986; Gibson et al., 1987; Takahashi et al., 1990). Furthermore, the mAbs UJ13A and T-199 stain medulloblastomas, neuroblastomas, and gliomas, but they also react with normal brain tissue (Allan et al., 1983; Feickert et al., 1989). In contrast, the mAbs C12 and D12 react with the cytoplasm or cell membranes of gliomas and medulloblastomas, but not with neuroblastomas and normal brain tissue (Wikstrand et al., 1986). The molecular weights of the antigens detected by C12 and D12 are 180 kDa and 88 kDa respectively.

The above findings suggest that the ONS-M21 antigen is a new tumour associated-antigen which is specifically expressed on the cell membranes of medulloblastomas, PNETs, and gliomas.

Bailey and Cushing described medulloblastomas as being derived from medulloblasts, the precursor cell of neuroblasts and glioblasts (Bailey & Cushing, 1925). However, most atuhors have reported that medulloblastomas express only a neuronal phenotype. In contrast, the results of the present study indicate that medulloblastomas may share common neuroectodermal elements with gliomas, or else that medulloblastomas may have either a neuronal or a glial phenotype.

Since ONS-M21 reacts with medulloblastomas, PNETs, and gliomas, it may be useful in their differential diagnosis from other brain tumours. In addition, the strong reactivity of ONS-M21 with these brain tumours and its lack of reactivity with normal brain tissue may also allow its clinical application. Since ONS-M21 is an mAb of the IgG1 subclass, its clinical application for conjugation to drugs, radionuclides, or toxins, is theoretically feasible.

The authors wish to thank Dr M. Tsudo for his technical advice, and also wish to thank Dr M. Nakayama (Osaka Medical Center and Research Institute for Meternal and Child Health) for providing most of the neuroblastoma and fetal brain tissue samples.

### References

ALLAN, P.M., GARSON, J.A., HARPER, E.I., ASSER, U., COAKHAM, H.B., BROWNELL, B. & KEMSHEAD, J.T. (1983). Biological characterization and clinical applications of a monoclonal antibody recognizing an antigen restricted to neuroectodermal tissues. *Int. J. Cancer*, **31**, 591–598.

BAILEY, P. & CUSHING, H.L (1925). Medulloblastoma cerebelli: A common type of midcerebellar glioma of childhood. *Arch. Neurol. Psychiatry*, **14**, 192–224.

COOMBES, R.C., BUCKMAN, R., FORRESTER, J.A., SHEPHERD, V., O'HARE, M.J., VINCENT, M., POWLES, T.J. & NEVILLE, A.M. (1986). *In vitro* and *in vivo* effects of a monoclonal antibody-toxin conjugate for use in autologous bone marrow transplantation for patients with breast cancer. *Cancer Res.*, **46**, 4217–4220.

DEHNER, L.P. (1986). Peripheral and central primitive neuroectodermal tumors. *Arch. Pathol. Lab. Med.*, **110**, 997–1005.

FARWELL, J.R., DOHRMANN, G.J. & FLANNERY, J.T. (1984). Medulloblastoma in childhood: an epidemiological study. *J. Neurosurg.*, **61**, 657–664.

FEICKERT, H.J., PIETSCH, T., HADAM, M.R., MILDENBERGER, H. & RIEHM, H. (1989). Monoclonal antibody T-199 directed against human medulloblastoma: characterization of a new antigenic system expressed on neuroectodermal tumors and natural killer cells. *Cancer Res.*, **49**, 4338–4343.

GROSS, N., BECK, D., CARREL, S. & MUNOZ, M. (1986). High selective recognition of human neuroblastoma cells by mouse monoclonal antibody to a cytoplasmic antigen. *Cancer Res.*, **46**, 2988–2994.

GIBSON, F.M. & KEMSHEAD, J.T. (1987). A monocloncal antibody (FMG25) that can differentiate neuroblastoma from other small round-cell tumours of childhood. *Int. J. Cancer*, **39**, 554–559.

HERSHATTER, B.W., HALPERIN, E.C. & COX, E.B. (1986). Medulloblastoma: the Duke University Medical Center Experience. *Int. Radiat. Oncol. Biol. Phys.*, **12**, 1771–1777.

HE, X., SKAPEK, S.X., WIKSTRAND, C.J., FRIEDMAN, H.S., TROJANOWSKI, J.Q., KEMSHEAD, J.T., COAKHAM, H.B., BIGNER, S.H. & BIGNER, D.D. (1989). Phenotypic analysis of four human medulloblastoma cell lines and transplantable xenografts. *J. Neuropathol. Exp. Neurol.*, **48**, 48–68.

HE, X., WIKSTRAND, C.J., FRIEDMAN, H.S., BIGNER, S.H., PLEASURE, S., TROJANOWSKI, J.Q. & BIGNER, D.D. (1991). Differentiation characteristics of newly established medulloblastoma cell lines (D384Med, D425Med, and D458Med) and their transplantable xenografts. *Lab. Invest.*, **64**, 833–843.

JONES, D., FRITSCHY, J., GARSON, J., NOKES, T.J.C., KEMSHEAD, J.T. & HARDISTY, R.M. (1984). A monoclonal antibody binding to human medulloblastoma cells and to the platelet glycoprotein IIb-IIIa complex. *Br. J. Hematol.*, **57**, 621–631.

JENNINGS, M.T., JENNINGS, V.D.L., ASADOURIAN, L.L.H., ROSENBLUM, M., ALBINO, A.P., CAIRNCROSS, J.G. & OLD, L.J. (1989). Five novel cell surface antigens of CNS neoplasms. *J. Neurol. Sci.*, **89**, 63–78.

KEMSHEAD, J.T., FRITSCHY, J., GARSON, J.A., ALLAN, P., COAKHAM, H., BROWN, S. & ASSER, U. (1983). Monoclonal antibody UJ 127:11 detects a 220,000–240,000 kdal. glycoprotein present on a subset of neuroectodermally derived cells. *Int. J. Cancer*, **31**, 187–195.

LEONG, A.S.-Y. & MILIOS, J. (1987). Atypical fibroxanthoma of the skin: a clinicopathological and immunohistochemical study and a discussion of its histogenesis. *Histopathology*, **11**, 463–475.

MORIUCHI, S., SHIMIZU, K., YAMADA, M., MABUCHI, E., TAMURA, K., PARK, K.C. & HAYAKAWA, T. (1991). Cytotoxic effects of a new antitumor antibiotic, FK973, in malignant glioma. *Anticancer Res.*, **11**, 2079–2084.

OGAWA, A., SUGIHARA, S., NAKANISHI, Y., SUZUKI, S., SASAKI, A., HIRANO, J. & NAKAZATO, Y. (1990). Intermediate filament expression in non-neoplastic pituitary cells. *Virchows Arch. B*, **58**, 331–340.

OKAMOTO, Y., MINAMOTO, S., SHIMIZU, K., MOGAMI, H. & TANIGUCHI, T. (1990). Interleukin 2 receptor beta chain expressed in an oligodendroglioma line binds interleukin 2 and delivers a growth signal. *Proc. Natl Acad. Sci. USA*, **87**, 6584–6588.

PASTAN, I. & GOTTESMAN, M. (1987). Multiple drug resistance in human cancer. *N. Engl. J. Med.*, **316**, 1388–1393.

RORKE, L.B. (1983). The cerebellar medulloblastoma and its relationship tc primitive neuroectodermal tumors. *J. Neuropathol. Exp. Neurol.*, **42**, 1–15.

RORKE, L.B., GILLES, F.H., DAVIS, R.L. & BECKER, L.E. (1985). Revision of the World Health Organization Classification of brain tumors for childhood brain tumors. *Cancer*, **56**, 1869–1886.

RAYMOND, W.A. & LEONG, A.S.Y. (1990). Oestrogen receptor staining of paraffin – embedded breast carincomas following short fixation in formalin: a comparison with cytosolic and frozen section receptor analyses. *J. Pathol.*, **160**, 295–303.

REED, J.C., MEISTER, L., TANAKA, S., CUDDY, M., YUM, S., GEYER, C. & PLEASURE, D. (1991). Differential expression of *bcl2* protooncogene in neuroblastoma and other human tumour cell lines of neural origin. *Cancer Res.*, **51**, 6529–6538.

TROJANOWSKI, J.Q., FRIEDMAN, H.S., BURGER, P.C. & BIGNER, D.D. (1987). A rapidly dividing human medulloblastoma cell line (D283MED) expresses all three neurofilament subunits. *Am. J. Pathol.*, **126**, 358–363.

TACHIBANA, I., WATANABE, M., TANIO, Y., HAYASHI, S., HOSOE, S., SAITO, S., MATSUNASHI, M. OSAKI, T., SHIGEDO, Y., MASUNO, T. & KAWASE, I. (1992). Generation of a small cell lung cancer variant resistant to lymphokine-activated killer (LAK) cells: association with resistance to a LAK cell-derived, cytostatic factor. *Cancer Res.*, **52**, 3310–3316.

TAKAHASHI, H., BELSER, P.H., ATKINSON, B.F., SELA, B.A., ROSS, A.H., BIEGEL, J., EMANUEL, B., SUTTON, L., KOPROWSKI, H. & HERLYN, D. (1990). Monoclonal antibody-dependent, cell-mediated cytotoxicity against human malignant gliomas. *Neurosurg.*, **27**, 97–102.

TAMURA, K., SHIMIZU, K., YAMADA, M., OKATOMO, Y., MATSUI, Y., PARK, K.C., MABUCHI, E., MORIUCHI, S. & MOGAMI, H. (1989). Expression of major histocompatibility complex on human medulloblastoma cells with neuronal differentiation. *Cancer Res.*, **49**, 5380–5384.

TANIO, Y., WATANABE, M., INOUE, T., KAWASE, I., SHIRASAKAh, T., IKEDA, T., HARA, H., MASUNO, T., SAITO, S., KAWANO, K., KITAMURA, H., KUBOTA, K., KODAMA, N., KAWAHARA, M., SAKATANI, N., FURUSE, K., YAMAMOTO, S. & KISHIMOTO, S. (1990). Chemoradioresistance of small cell lung cancer cell lines derived from untreated primary tumors obtained by diagnostic bronchofiberscopy. *Jpn. J. Cancer Res.*, **81,** 289–297.

WIKSTRAND, C.J., MCLENDON, R.E., BULLARD, D.E., FREDMAN, P., SVENNERHOLM, L. & BIGNER, D.D. (1986). Production and characterization of two human glioma xenograft-localizing monoclonal antibodies. *Cancer Res.*, **46,** 5933–5940.

WAKABAYASHI, T., YOSHIDA, J., SEO, H., KAZO, K., MURATA, Y., MATSUI, N. & KAGEYAMA, N. (1988). Characterization of neuroectodermal antigen by a monoclonal antibody and its application in CSF diagnosis of human glioma. *J. Neurosurg.*, **68,** 449–455.

ZIMMERMANN, U. (1982). Electric field-mediated fusion and related electrical phenomena. *Biochem. Biophys. Acta*, **694,** 227–277.